# Quality assurance and quality control for human biomonitoring data—focus on matrix reference materials

**DOI:** 10.1007/s00216-025-05859-3

**Published:** 2025-04-22

**Authors:** Đorđe Tadić, Ana Pires de Lima, Marina Ricci

**Affiliations:** European Commission, Joint Research Centre (JRC), Geel, Belgium

**Keywords:** Human biomonitoring, Certified reference materials, Quality assurance and quality control, Harmonization, Proficiency testing, Per- and polyfluoroalkyl substances

## Abstract

Human biomonitoring (HBM) is an essential tool for making evidence-based policy decisions upon assessment of human exposure to pollutants. In contrast, the heterogeneity of data reliability across studies has been identified as a weakness in current HBM research. The scientific community is currently encountering measurement challenges due to gaps in the availability of quality assurance/quality control (QA/QC) tools. This article provides a summarised view on the availability of reference materials in human matrices such as hair, serum, blood, plasma, urine, and breast milk for selected groups of organic contaminants (e.g., pesticides, flame retardants, perfluorinated compounds, perchlorate, phthalates, phenols) and elements (e.g., mercury, arsenic, cadmium, chromium, lead, nickel, tin) of relevance to human health. The QA/QC situation of HBM measurement data with regard to laboratories’ performance in proficiency testing schemes is also addressed, highlighting areas for improvement. Finally, the article uses the example of per- and polyfluoroalkyl substance analysis to illustrate QA/QC challenges specifically related to the HBM field.

**Graphical Abstract:**

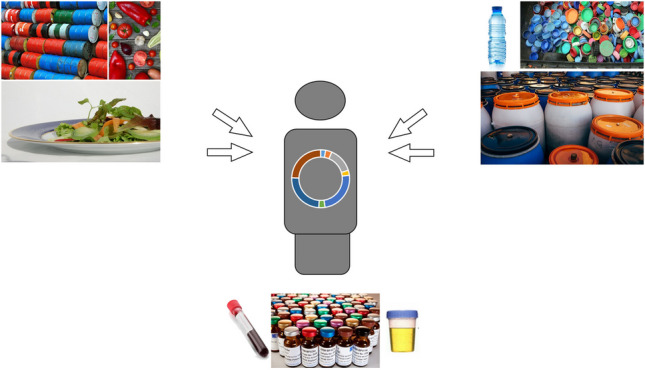

**Supplementary Information:**

The online version contains supplementary material available at 10.1007/s00216-025-05859-3.

## Introduction

Regulations that aim to protect from the risks posed by chemicals are of utmost importance in a society where global chemical industry surpassed $5 trillion in 2017 and is projected to double by 2030. To address these issues, the Global Chemicals Outlook II, mandated by the United Nations Environmental Assembly, seeks to alert policymakers and other stakeholders about the critical role of the sound management of chemicals and waste in sustainable development [1]. In the European Union (EU), where the chemical sector is the fourth-largest manufacturing producer [2], the Chemicals Strategy for Sustainability Towards a Toxic-free Environment is part of the European Green Deal and its Zero Pollution ambition [3].

The interdependency of human health and the environment is recognised in the EU One Health approach, a comprehensive strategy for better well-being. To translate these ideas into action and improve understanding of the relationship between stressors, environmental pollution, and health, contributions from human biomonitoring (HBM) studies are essential [4, 5]. HBM studies conducted in limited geographical areas (generally country-based) such as the German Environmental Survey (GerES) [6], the Czech Environmental Health Monitoring System [7], the French National Biomonitoring Programme [8], the Italian PROBE [9], the Spanish BIOAMBIENT.ES [10], and the Flemish Human Biomonitoring Studies [11], and focused on specific groups of contaminants, demonstrated the need for a more comprehensive overview. The European Commission, through the European Environment and Health Action Plan 2004–2010 [12], launched in 2009 the twin projects COPHES/DEMOCOPHES, which laid the foundations for future more coherent actions on HBM [13]. The Human Biomonitoring for Europe Joint Project (HBM4EU, 2017–2021) [14] was funded under Horizon 2020 to inform the policymaking process and further develop chemical exposure science. Its follow-up, the large-scale 7-year initiative “Partnership for the Assessment of Risks in Chemicals (PARC)” [15], was launched in 2022 with the main goal of developing next generation chemical risk assessment methods and supporting EU and national strategies with new data, knowledge, networks and skills to reduce risks posed by hazardous chemicals to health and the environment. The need for harmonization of HBM results on a global scale has been evaluated and the heterogeneity of data and limitations in quality assurance across studies have been identified as a weakness of current HBM studies that has to be addressed [5, 16, 17]. To address the emerging challenges, the PARC project has a specific task within the Working Group 4 dedicated to quality assurance/quality control (QA/QC). Data heterogeneity among HBM studies can be caused by various factors, including study design in the pre-analytical phase, and harmonization of applied protocols within projects has often become a specific task or deliverable.

Reliable data are necessary not only for comparison among laboratories’ performances but also to enable the identification and tracking of time trends that could be useful for designing and implementing public health policies. There are three main QA/QC pillars that can help improving the comparability and reliability of analytical data: application of validated, standard and/or reference methods, participation to proficiency testing (PT) schemes, and use of (certified) reference materials (CRMs). Accreditation mainly rests on these pillars. These pillars are neither individually sufficient nor completely independent in ensuring accuracy of results. It is well recognised the chicken-and-egg dilemma of properly validated analytical methods that are necessary for the certification of reference materials while CRMs are needed for a full method validation. When one or both of these two pillars are not available and/or fully developed, the only way towards a fully deployed QA/QC system is by incremental advancements. PT schemes are fundamental in this situation where the lack of one component hinders the development of the other and certainly foster harmonization, being sometimes the only way to start the quality journey. However, consensus values are generally not metrologically traceable, leaving a question mark on their trueness. The HBM community is currently facing challenges related to a limited number of established reference/standard methods and a lack of relevant CRMs (especially in matrix). As a result, QA/QC efforts rely mainly on inter-laboratory comparisons, which often require more than one round of testing for uplifting laboratories’ performance [18].

For what concern standard and reference methods, the Joint Committee for Traceability in Laboratory Medicine database lists only reference methods for metals, i.e., cadmium in blood and urine, mercury in urine and blood, lead in urine and blood, and cobalt in urine [19]. Current International Organization for Standardization (ISO) methods for metals, organochlorine pesticides, and polychlorinated biphenyls (PCBs) in milk, milk products, and infant formulas can provide help in breast milk analysis [20–22].

In this contribution, we mainly focused on the availability of matrix (C)RMs for HBM, but we also looked into the HBM PT schemes landscape. Reference and standard methods are so few that they deserved only the brief mention reported above in the text. As CRMs producers, we wanted to “map” the field in order to identify gaps and needs in analyte/matrix combinations in which to concentrate our development and production efforts in. We therefore considered six major HBM programmes (the EU PARC [15], the Canadian Health Measures Survey (CHMS) [23], the United States National Health and Nutrition Examination Survey (NHANES) [24], the Korean National Environmental Health Survey (KoNEHS) [25], the Japan Environment and Children’s Study (JECS) [26–31], and the China National Human Biomonitoring (CNHBM) [32]) providing a comprehensive overview of the biomarkers included therein and correlated available matrix CRMs. Together with the critical evaluation of the outcomes of these searches, we look into specific challenges in the production of CRMs suited to biomonitoring studies and their limitations.

The example of per- and polyfluoroalkyl substances (PFAS) analysis in HBM serves to illustrate the numerous and significant challenges that the scientific community is currently facing in their determination, as well as the difficulties encountered by RM producers. This example is particularly relevant and representative for the HBM field as it also encompasses emerging and novel applications in methods and ways of monitoring contaminants in humans that still require validation and incorporation into standard procedures (e.g., dried blood spot, non-target and suspected screening).

## Methods

Regarding the availability of CRMs, we conducted a search in several open-access databases (Table 1) using the following keywords: human blood, urine, serum, plasma, hair, and nails, and combination thereof. These databases provide a general overview, but the information found is not always up-to-date or complete. Therefore, we additionally consulted numerous producers and distributors’ websites (Table 1). We included producers/distributors of non-certified RMs (including PT organisers, selling the leftover test materials from PT schemes) as laboratories in the clinical chemistry field widely and commonly rely on those.

**Table 1:** Screened RMs databases and producers/distributors

Databases for RMs	
CNRM	National Sharing Platform for Reference Materials
COMAR	Code of Reference Materials
EVISA	European Virtual Institute for Speciation Analysis
JCTLM	Joint Committee for Traceability in Laboratory Medicine
KCDB (BIPM)	Key Comparison database of the International Committee for Weights and Measures
LGC	LGC Standards
RMInfo	Reference Materials Total Information Services in Japan
CNRM	National Sharing Platform for Reference Materials
Producers/distributors of RMs	
Bio-Rad	Bio-Rad Laboratories, USA
CIL	Cambridge Isotopes Laboratories, USA
CTQ	Centre de Toxicologie du Québec, Canada
CERI	Chemical Evaluation and Research Institute, Japan
BAM	Federal Institute for Materials Research and Testing, Germany
IAEA	International Atomic Energy Agency, Analytical Quality Control Services, Austria
JRC	Joint Research Centre of the European Commission, Belgium
KRISS	Korea Research Institute for Standards and Science, South Korea
LGC	LGC Standards, UK
Medichem Diagnostica	Medichem Diagnostica, Germany
Mintek	Mintek, South Africa
NCS	National Analysis Centre for Iron and Steel, China
NIES	National Institute for Environmental Studies, Japan
NIOHP	National Institute for Occupational Health and Poisoning Control, Chinese Center for Disease Control and Prevention, China
NIM	National Institute of Metrology, China
NIMD	National Institute of Minamata Disease, Japan
NIST	National Institute of Standards and Technology, USA
LNE	National Laboratory of Metrology and Testing, France
CENAM	National Metrology Center, Mexico
NMIJ	National Metrology Institute of Japan, Japan
UME	National Metrology Institute-TÜBITAK, Turkey
NRC-CNRC	National Research Council Canada, Canada
RECIPE	RECIPE, Germany
SERO	SERO, Norway
UTAK	UTAK Laboratories, USA
WL	Wellington Laboratories Inc., Canada

The search for HBM PT schemes was carried out via the European PT Information System (EPTIS) database using the same keywords as for CRM search [33]. Additionally, PT rounds organised as strategic objectives of HBM programmes were also included.

For the purpose of a systematic review of biomarkers, a subset of HBM programmes was selected based on predefined inclusion criteria. The primary inclusion criteria for programme selection for the purpose of this review were the availability of detailed information regarding the rationale behind biomarkers selection, a comprehensive list of selected biomarkers, and future perspectives and outcomes. A search was conducted to identify relevant information through websites, scientific literature, and governmental or funding body websites. This systematic search enabled the identification of six main HBM programmes providing global coverage: PARC [15], CHMS [23], NHANES [24], KoNEHS [25], JECS [26–31], and CNHBM [32]. To identify gaps in CRM availability and prioritise the development of new CRMs (section “Finding the gaps and flagging the needs in HBM”), we started by considering the biomarkers list from the PARC programme. Additionally, we identified common biomarkers that were consistently measured across at least three of the selected HBM programmes, allowing us to focus on the most frequently analysed biomarkers and assess the need for new CRMs. This list finally included 17 elements (aluminium, arsenic and its compounds, cadmium, chromium, cobalt, copper, lead, lithium, manganese, mercury, molybdenum, nickel, platinum, selenium, strontium, tin, and zinc) and 68 organic compounds (e.g., pesticides, PFAS, phthalates and their substitutes, and tobacco alkaloids) (Supplementary Material – Biomarkers summary). As evidence of the relevance of the selected elements in the context of HBM studies, inclusion in lists of public health concern [34] and in regulatory frameworks were considered [35–37].

The biomarkers selection was further critically evaluated by examining the laboratories’ performances in the PT schemes: the poorer the general performance for a specific analyte/matrix combination, the greater the need for quality control tools such as CRMs.

Presentation and visualization of results were made with MsExcel® and R version R- 4.4.0 (2024–04–24) using CRAN Package circlize: Circular Visualization (https://cran.r-project.org/web/packages/circlize/index.html).

## Biomarkers for HBM

The selection of biomarkers for HBM programmes is a critical step that involves multiple factors, in addition to considering main priority pollutants monitored in the environment. A review of the selection criteria used in HBM studies in the EU [38], Korea [39], the USA [40], and Canada [23] reveals three main pillars: health-related, feasibility-related, and policy-related criteria. These criteria ensure that the selected biomarkers are relevant to human health, can be measured accurately, and provide valuable information for public health policy development. Policy-related criteria consider the broader policy context, including national and international treaty commitments, as well as current and anticipated health policy actions.

**Fig. 1:**
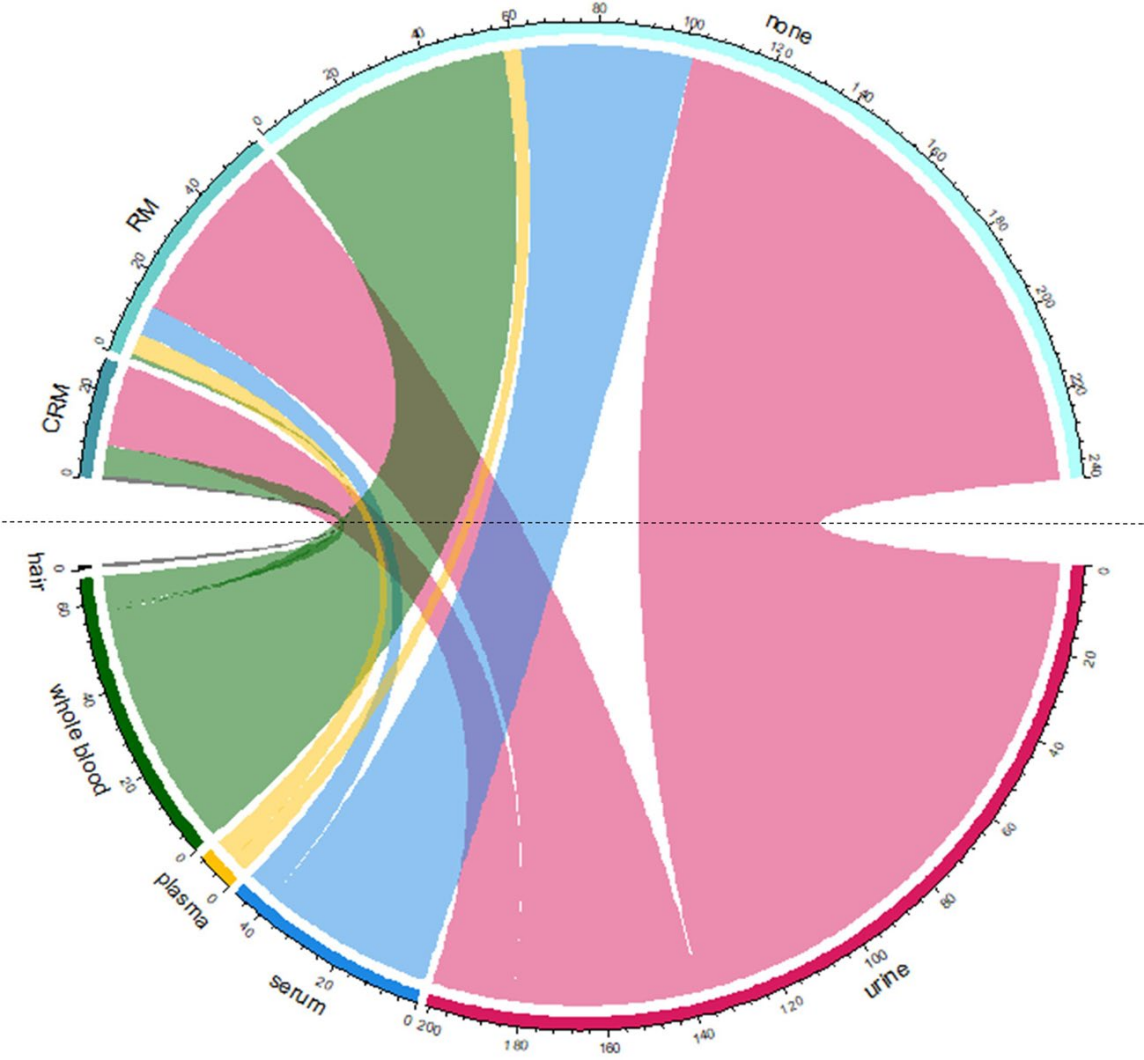
Numbers of biomarkers, from selected HBM programmes, analysed in different matrixes (i.e., hair, whole blood, plasma, serum, and urine) are presented in the lower part of the figure, whereas the availability of RMs (i.e., CRM, RM, and none) for selected biomarker-matrix combinations is shown in the upper part of the figure

The selected HBM programmes (conducted in the EU, Canada, Japan, China, Korea, and the USA) yielded a comprehensive dataset of 324 biomarkers, providing a representative dataset for in-depth analysis. A summary of selected biomarkers from the following programmes: PARC (2021–2027), CHMS Cycle 6 (2018–2019), NHANES (2017–2018), KoNEHS (2018–2020), JECS (2011–2027), and CNHBM (2017–2018) is presented in Fig. 1 (Supplementary Material – Biomarkers summary). The data show that the most frequent analytes in these six HMB programmes are elements and their compounds, pesticides, phthalates, bisphenols, and parabens. Figure 1 shows that urine is the matrix with the highest number of contaminants analysed. This is likely due to the minimal ethical constraints and the non-invasive nature of urine sampling. Urine can be easily collected from a large number of participants, particularly when targeting children (for example, NHANES and GerES). Therefore, urine is the primary and most commonly used matrix in HBM studies, particularly for evaluation of recent exposure. Specifically, 201 chemical compounds are listed as biomarkers for analysis in urine. Notably, RMs are available for only 30% of these compounds.

CRMs are available only for 11 elements, two neonicotinoid pesticides, perchlorate, and five PAH metabolites accounting for only 9% of the 201 biomarkers. However, several groups of contaminants lack commercially available (C)RMs, such as pyrethroid pesticides, organochlorine pesticides, glyphosate and aminomethylphosphonic acid, phthalate substitutes, tobacco-related compounds, and organophosphate and brominated flame retardants, among others. Additionally, only one RM is available for bisphenol A, despite 16 bisphenol-related compounds being analysed in urine.

The second matrix in terms of the number of analysed contaminants/biomarkers is whole blood, with 36 volatile organic compounds (VOCs) and 23 elements being analysed. (C)RMs are available only for eight elements, and no (C)RMs are available for biomarkers such as acrylamide and its metabolite, ethylene oxide haemoglobin adduct, disinfection by-products, and VOCs. In serum, the most typical analysed biomarkers are the PFAS, consisting of 45 individual compounds. Only seven individual PFAS compounds (of the 45) can count on one available RM in serum (SRM 1957) from National Institute of Standards and Technology (NIST). Analysis of plasma includes nine PFAS and the only RM currently available for six of these PFAS compounds is SRM 1950. Finally, mercury measurement in hair is included in PARC, and both RMs and CRMs are available on the market.

## Matrix RMs for HBM

In this survey, we primarily included CRMs, but we also considered non-certified materials due to the limited availability of CRMs in human matrices. The obtained data are presented in the Supplementary Material – CRMs summary. We have identified 48 commercially available materials, categorised into six human matrices i.e., breast milk, blood, serum, plasma, urine, and hair, based on the criteria outlined below. Although no materials were identified for the saliva matrix, it is still included in this section to ensure completeness.

Matrix RMs can contain varying numbers (*n*) of biomarkers (ranging from a few to over a hundred) and concentration levels. For example, NIST provides a series of RMs with a high number of biomarkers, both certified and non-certified, including non-fortified and fortified materials, such as SRM 1953 (*n* = 65) and SRM 1954 (*n* = 93) for milk, SRM 3672 (*n* = 52) and SRM 3673 (*n* = 50) for urine, and SRM 1957 (*n* = 57) and SRM 1958 (*n* = 105) for serum.

**Fig. 2:**
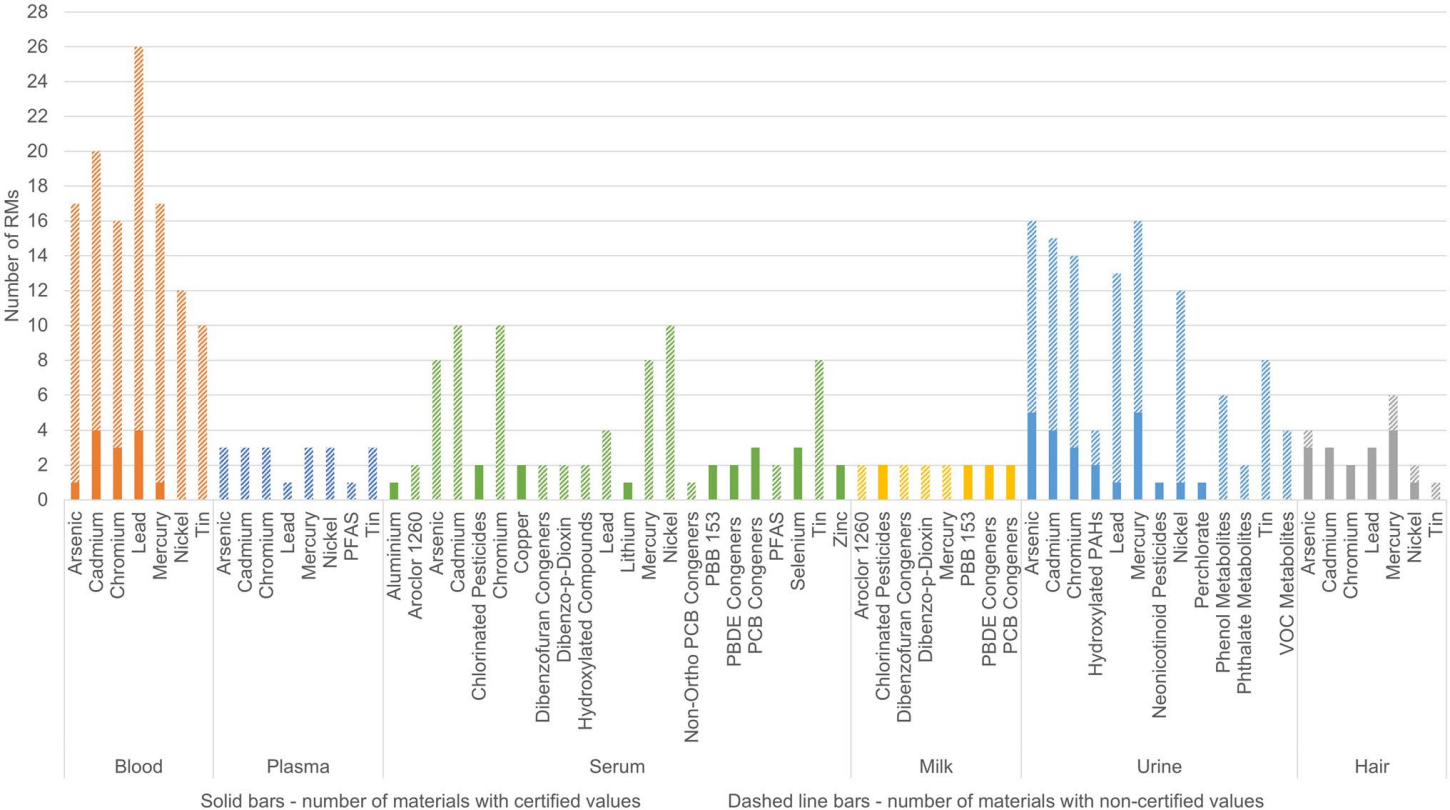
Availability of certified material with at least one certified value per biomarker group (solid bars) and non-certified (dashed lines bars) materials for different biomarker-matrix combinations

In displaying Fig. 2, we followed certain criteria. A material is considered certified (represented by solid bars in Fig. 2) if it has at least one certified value, even if non-certified values are assigned for other biomarkers. This distinction is made to acknowledge that some materials have both certified and non-certified values. RMs presenting different levels of the same biomarker in the same matrix were counted as single (C)RM. Figure 2 summarises the available certified and non-certified materials for biomarker group-matrix combinations. A clear trend can be observed, with elements and their compounds exhibiting a higher number of materials that offer more than five options (the only exception for organic compounds being phenol metabolites in urine). Non-certified values are more prevalent in these materials.

### Breast milk

Human milk offers a non-invasive and very cost-effective sampling method, and its higher lipid content facilitates the detection of lipophilic compounds compared to other matrixes like blood serum [41]. The use of this matrix provides additional value by offering insights into possible exposure of the breastfed infant. On the other hand, one obvious drawback is the focus on a narrow population group.

NIST is the only producer found to offer CRMs in human breast milk. SRM 1953 and its fortified version, SRM 1954, report several certified values for PCB congeners, polybrominated diphenyl ether (PBDE) congeners, chlorinated pesticides (i.e., hexachlorobenzene, oxychlordane, hexachlorocyclohexane mirex, dichlorodiphenyldichloroethylene, chlordane, nonachlor, dichlorodiphenyldichloroethane, dichlorodiphenyltrichloroethane), and PBB- 153. Reference values are also available for dibenzofuran congeners, dibenzo-p-dioxins, aroclor 1260, and mercury (Supplementary Material – CRMs summary Table S1).

Cow’s milk and infant formulas CRMs can be used as proxy matrices for breast milk analysis [42, 43]. The applicability of a proxy matrix has to be evaluated vs the specific analytical methodology employed as matrix differences might impact the measurement result. For example, the robustness of a method towards variations in lipid content can influence the suitability of skimmed cow’s milk for use as a proxy for raw human milk. For instance, ERM-BD150, BCR- 607, BCR- 450, and BCR- 188 from the Joint Research Centre (JRC) can be used for the analysis of elements, dioxins/furans, PCBs, and pesticides, respectively. However, these materials were excluded from the summary because they are not human matrices.

### Blood

To the extent of our knowledge, there are no RMs for organic contaminants in whole human blood. In contrast, we identify several certified and non-certified materials for inorganic contaminants screened in this survey. Whole blood CRMs for inorganic elements are offered by different producers (NIST, National Institute of Minamata Disease (NIMD), JRC, and LGC) as well as non-certified RMs (RECIPE, SERO, Bio-Rad, and UTAK) (Supplementary Material – CRMs summary Table S5). It is worth mentioning that LGC 8276 was produced by spiking equine blood with the elements of interest (i.e., chromium, nickel, cobalt, and molybdenum). Given its proven commutability with human blood using the specified methods of analysis as reported in the CRM certificate, it was included in this survey. BCR- 634, BCR- 635, and BCR- 636 are certified for lead and cadmium at three increasing concentration levels. These JRC materials are almost exhausted but they are already replaced by new CRMs (ERM-DA634, ERM-DA635, and ERM-DA636) with an enlarged panel of certified elements [44]. NIST, NIMD, JRC, and LGC released CRMs with certified values for arsenic, cadmium, chromium, mercury and its compounds, lead, selenium, zinc, manganese, cobalt, and molybdenum. In addition, SERO and RECIPE offer non-certified materials with multiple concentration levels for all these elements, whereas Bio-Rad and UTAK materials cover a slightly smaller range of analytes. For nickel and tin, only non-certified materials are available.

### Serum

SRM 1957 and SRM 1958 from NIST offer certified values for PBDE congeners, PCB congeners, chlorinated pesticides, and PBB- 153. These two serum-based materials also provide reference values for dibenzofuran congeners, dibenzo-p-dioxins, perfluorinated compounds, hydroxylated compounds, and aroclor 1260. The values reported in SRM 1957 for PFAS match the experimental values found in HBM literature, demonstrating that the material is representative of the levels in the population [45].

In this matrix, few CRMs (HRM- 2011 A, GBW09152, SRM 956 d, and BCR- 637, BCR- 638, and BCR- 639) are available for inorganic contaminants, including copper, selenium, zinc, lithium, aluminium, and selenium. Several RMs are produced within the scope of ISO 13485 [46]. RMs from SERO offer the widest range of inorganic elements reported (including arsenic, cadmium, chromium, lead, mercury, nickel, and tin), alongside RECIPE and UTAK, which also provide materials for analysis in several levels (Supplementary Material – CRMs summary Table S4).

### Plasma

SRM 1950 (NIST) provides reference values for six perfluorinated compounds (as the total of both branched and linear forms of the analytes). This is the only plasma RM that provides values for organic compounds. Most PFAS analyses are performed in serum; however, some studies also analyse plasma and whole blood. Values of PFAS reported in previous studies in plasma generally match the levels found in SRM 1950 [47–50]. Regarding inorganic elements, RECIPE offers two RMs in lyophilised plasma for arsenic, cadmium, chromium, mercury, nickel, and tin. Recent studies have reported the presence of arsenic and cadmium in plasma (normal, maternal, and cord) [51, 52].

### Urine

NIST offers SRM 3672 and SRM 3673 that contain several certified values for hydroxylated polycyclic aromatic hydrocarbons (PAHs) and reference values for an extensive list of phenol metabolites, phthalate metabolites, and VOC metabolites. NIST also offers SRM 3668, which is available with two certified levels of perchlorate. NMIJ produced an artificial urine-based material for the measurement of neonicotinoid pesticides (NMIJ CRM 7408-a), with both certified (acetamiprid and thiamethoxam) and non-certified values. Concerning inorganic contaminants, urine is one of the most well-covered matrix in terms of the number of RMs, both certified and non-certified. Among the certified materials, there are products from at least two different producers for the analysis of arsenic and its species, cadmium, chromium, lead, and mercury. For the analysis of nickel and tin, only SRM 2668 is available, which includes both elements at two different levels, but only one certified value for Ni. Additionally, all these elements can be analysed at different levels in multiple urine clinical RMs offered by SERO, RECIPE, UTAK, and Bio-Rad. Phenolic compounds are present in two materials from RECIPE and two from Bio-Rad; these are the only non-certified RMs found for the organic environmental contaminants considered in this survey. In the CNRM database for materials produced in China, additional materials were found for the analysis of trace elements (GBW09102, GBW09103) and lead (GBW09104a and GBW09105a) in lyophilised human urine.

### Hair and nails

Hair is particularly used to assess toxic metal contamination [53, 54]. Several producers offer homogenised human hair materials certified for elements. GBW07601, originally produced more than 30 years ago (by the Shanghai Institute of Nuclear Research of the Chinese Academy of Sciences, Shanghai, China), was later replaced by its successor GBW07601a (GSH- 1a), which is still widely used due to its coverage of more than 60 inorganic elements. A few years later, the same producer released a similar material, GBW09101, which reports only half of the analytes and was later re-certified as GBW09101b. These materials, (GBW07601a (GSH- 1a)) and GBW09101b), were also issued by the China National Analysis Center for Iron and Steel, with adopted codes of NCS DC 73347a and NCS ZC 81002b, respectively. As a result, different identifications for the same material may appear in the literature. Released at the beginning of the century, the IAEA catalogue still includes the RMs IAEA- 085 and IAEA- 086 for the analysis of mercury, methylmercury, selenium, and zinc. In the past 10 years, two new materials for human hair have been released. ERM-DB001 from JRC provides certified values for arsenic, cadmium, lead, and mercury, while NIMD- 01 from NIMD was designed for the analysis of total mercury, methylmercury, copper, zinc, selenium, and arsenic (the latter reported as indicative value). NIES No.13, a human hair material, was recently discontinued but has already been replaced by its successor, NIES No.13a. Tehrani et al. [55] produced four candidate caprine horn RMs (NYS RMs 18–01 to 18–04) for trace element analysis, aiming to improve measurements in keratinised matrices rather than hair. Homogeneity and commutability were assessed, indicating that these materials are generally suitable for the analysis of human nails for eight elements (including chromium and lead).

### Saliva

Saliva can serve as an alternative non-invasive biospecimen for HBM, in addition to urine and breast milk. Sampling methodology for saliva is highly critical, and there are several ways of sampling, as well as instructions and guidelines on how to perform it (e.g., eating and drinking before, tooth brushing). Recent studies have analysed various contaminants in saliva, including PAHs, pesticides, bisphenols, parabens, and phthalates. The biggest challenge is the absence of a standardised sampling procedure and storage protocol [56]. Currently, saliva is not included in HBM studies, and no CRMs or RMs are available for this matrix.

## HBM PT schemes

For the past four decades, two PT schemes have operated alongside intended HBM programmes. Specifically, the external quality assessment schemes established in 1979 by the Centre de Toxicologie du Québec (CTQ) and the German External Quality Assessment Scheme (G-EQUAS) established in 1982 have provided QA for occupational and environmental toxicological analyses in human matrices. The list of biomarkers addressed by these schemes is updated regularly to reflect the latest developments and advances in the field. Evaluation of emerging scientific evidences, changes in regulatory requirements, advances in analytical technology as well as economic aspects come into play. CTQ schemes [57] cover a wide range of contaminants in relevant biomonitoring matrices (i.e., bisphenols, pesticides, hydroxy-PAHs, parabens, PFAS, and flame retardants in urine; PCBs, brominated flame retardants, organochlorine pesticides, PFAS in serum; elements in blood, serum and urine), whereas the latest round of G-EQUAS 74–2024 [58] includes metals, aromatic hydrocarbons, chlorinated hydrocarbons, solvents, amines and phenolic compounds, pesticides, plasticisers, mercapturic acids, tobacco-specific parameters, alcohols, and organohalogen compounds. In fact, G-EQUAS is being utilised as a QC tool in the PARC project, demonstrating the value of PT schemes in supporting research studies and for advancing relevant knowledge as basis for policy evaluations and opinions.

Additionally, PT schemes are organised to lay the foundation for future HBM programmes, with a strategic focus on capacity building of laboratory proficiency in a specific analysis. For instance, within the framework of the United Nations Environment Programme (UNEP) a project was implemented aimed at developing a global plan for mercury monitoring. Specifically, the project provided technical support to laboratories to establish and demonstrate their competences in mercury analysis [59]. Similarly, the HBM4EU programme included interlaboratory comparisons and troubleshooting initiatives as one of the key deliverables, with the objective of establishing a network of laboratories proficient in the analysis of a wide range of biomarkers, thereby facilitating the implementation of future HBM programmes.

Despite the inclusion of organic contaminants in the aforementioned PT schemes, our survey indicates that PT programmes for elements analysis are more prevalent. Eleven PT schemes can be found in the EPTIS database [33] for elements in urine, bovine serum, frozen human blood, and milk. There is a considerably lower number of available PT schemes for organic contaminants in human matrices: only one PT scheme in urine and three PT schemes (including PFAS) in milk and infant formulas (which serves as adequate proxy matrices for the analysis of breast milk).

## Finding the gaps and flagging the needs in HBM

At the forefront of a research line, due to the incomplete development of each of the mentioned QA/QC pillars (i.e., CRMs, analytical methods, and PT schemes), none of them can assume a dominant or pivotal role. They are closely interconnected with one another, as the development of one pillar is dependent on the advancement of the others. Furthermore, they are also intertwined with the biomarker selection process (for current and future HBM programmes), as well as with relevant legislation. Their common outcome, reliable measurement results that will lead ultimately to the protection of human health, is rooted in the cooperative links between these entities. Due to the nature of such a setup, these factors have a dynamic, nonlinear relationship that nevertheless should not prevent to reach the goal to establish metrological traceable anchor points for HBM data, which will be discussed from the perspective of a CRM producer.

One of the main criteria for selecting biomarkers for HBM studies is the availability of reliable analytical methods, whose validation ideally rests on suitable CRMs. This is the chicken-and-egg dilemma already mentioned: CRMs can drive the development and optimization of analytical methods, which in turn can inform the selection of biomarkers for future HBM studies. The evaluation of the laboratories’ performance in past HBM PT schemes, together with the biomarkers/matrices that are already included, or foreseen to be included in HBM studies, for which there are no available RM, can serve as an indication of which CRMs need to be targeted (which will be discussed later in the text).

Poor performances, indicated by high coefficient of variation (CV) among laboratories, suggest that for specific compounds and/or matrices, QA/QC measures are not sufficiently mature, further training and capacity building for the participating laboratories and CRMs are needed to improve analytical capabilities. For example, the results of a series of interlaboratory assessments on persistent organic pollutants (POPs), conducted in support of the UNEP global monitoring plan under the Stockholm Convention, shows that the overall analytical performance in human milk is still insufficient, despite the matrix being recognised as preferred for monitoring average levels of POPs exposure from the environment. Among different target compounds (PCBs, organochlorine pesticides, PBDEs, one polybrominated biphenyl, and hexabromocyclododecane diastereomers), only few analytes achieved a between-lab CV value below 25%, even when present in relatively high concentrations, such as PCBs or p,p′-DDE [60]. Several factors were identified as contributing to this poor performance, including inadequate instrumentation, poorly developed extraction and clean-up protocols, and insufficient personnel training. These challenges are often linked to geographical disadvantages and inequalities, commonly faced by developing countries, and can hinder access to essential resources and services, such as instrument maintenance and support [60]. Across several comparison exercises, it has been observed that the human milk is often the least represented in terms of the number of participating laboratories and has the poorest analytical performance compared to other matrices [61]. Several studies have reported a lack of interlaboratory reliability when measuring specific elements in hair and other hard keratinised tissues [62, 63]. Among the most frequent reasons for unsuccessful results, the authors refer interlaboratory differences in washing procedures [64, 65], which are related to sample pre-treatment issues. Poor correlation with data from traditional biomonitoring matrices, such as blood, and environmental matrices (e.g., dust) as external sources of contamination has been reported [63, 66–68]. As presented in the summary of analysed biomarkers, hair is not the preferred matrix for assessing contaminants in biomonitoring studies, but it remains relevant for toxicological and forensic purposes, particularly for the analysis of analysing toxic metals. Results from UNEP/The Global Environment Facility interlaboratory study show that, despite an overall improvement over the past 10 years, about 80% of laboratories worldwide still do not have the necessary experience to analyse a large number of POPs and cannot generate globally comparable and reliable data. Challenging is also the fact that along the four rounds of interlaboratory assessment, new POPs have continually been added to the list as for example polychlorinated naphthalenes and short-chain chlorinated paraffins are planned to be included in the next round [69]. It has been shown that sharing of a RM and conducting a PT round can shed light on pitfalls in the analytical methods applied, such as background contamination, chromatographic separation of interfering and co-eluting peaks, selection of the most appropriate mass-to-charge ratio transitions for quantification, importance of the purity of enzyme used in the sample preparation, and selection of the internal standards [70]. It is often possible to purchase PT materials remaining from exercises as non-certified materials (e.g., from the CQT programme). These materials have a designated value and an acceptable uncertainty assigned for the analytes of interest. However, they lack long-term assessment of stability or other parameters/requirements (like the metrological traceability of the assigned value) necessary to confer them the status of CRM. Even though their availability is often limited to a reduced number of samples, one of the main advantages lies on the very competitive prices. For elements, as already mentioned, there are well established analytical methods, hence it is not a surprise that recent interlaboratory comparisons revealed overall positive laboratory performance in measuring chromium, arsenic, cadmium, mercury, and lead in human matrices such as urine, plasma, and whole blood [71–73].

Similar to the availability of PT schemes, CRMs for heavy metals in human matrices are widely available in the market, in contrast to CRMs for organic environmental contaminants. For the analysis of each element, there are CRMs available in at least three different human matrices (hair, blood, and urine), which overall cover a good range of concentrations (Supplementary material – CRMs summary Tables S3.2, S5, and S6). CRMs for organic contaminants within the scope of the paper are mainly produced by NIST, which includes two CRMs in human milk, one in plasma, three in urine and two in serum. Apart from NIST, NMIJ offers the only other two CRMs for biomonitoring, namely NMIJ CRM 7407-a in serum and NMIJ CRM 7408-a in artificial urine. Urine control/calibrator materials for some phenolic compounds are available from RECIPE and SERO, as listed in the Supplementary material – CRMs summary Table S3.1. As already highlighted, multiple factors enter into the ranking of parameters to be included in HBM programmes. This makes it challenging for RMs producers to predict which compounds should be prioritised. There are many directions in which CRM producers could stream their projects. Biomarkers that are always included in HBM studies and for which there are no available CRMs provide clear guidance for CRM producers on the current needs of the community. On the other hand, this conclusion needs to be paired with the current technological and analytical capabilities for producing the needed CRM. For example, in case of PARC, there are several groups of biomarkers that have been measured, but for which there are no available (C)RMs. More specifically, there are no available (C)RMs for five phthalate substitutes measured in urine (Supplementary material – Biomarkers summary). Only one CRM is available for acetamiprid in urine, whereas for other groups of organic contaminants, such as PFAS, phenols, phthalates, tobacco alkaloids, and some pesticides, there are only RMs available on the market. A notable observation emerges when examining the frequency of specific biomarkers in selected HBM programmes (Supplementary material – Biomarkers summary). We identified six organophosphate pesticides that are included in at least three HBM programmes worldwide, but lack both CRMs and RMs. Furthermore, our analysis reveals that for other frequently analysed biomarker groups, including PFAS, phenols, phthalates, tobacco alkaloids, and VOC metabolites, only RMs are available. All the above could provide hints to CRM producers on the gaps to bridge in HBM research. In this context, the authors would like to acknowledge the pioneering approach developed by the PARC initiative, which has compiled a list of potential upcoming biomarkers with a lack of QA/QC, known as PARC Discovery list [74]. This compilation also provides valuable insight for CRM producers, shedding light on the need for future CRM development and enabling them to prioritise their efforts accordingly.

In summary, a range of individual and collective actions (developing standard/reference methods, implementing in-house validation protocols, establishing PT schemes, selecting biomarkers and matrices for HBM studies, providing training and expertise, producing test RMs, developing candidate CRMs, publishing expert opinions on exposure, identifying key areas for funding, production of analytical reference standards, etc.) drives progress in the global context. High interdependence might slow down the whole process of establishing measures for human health protection. A network-organised approach, as seen in the PARC framework, enables various individual nodes in the network to exchange information and amplify their interactions, so that each of them can extract necessary information and improve its own capabilities.

One of the challenges in the production of CRMs for HBM lies in integrating clinical and environmental/food contaminants analysis, which requires laboratories’ expertise in a niche position between these two fields. This is especially true for the JRC, which relies on measurements from external laboratories for the value assignment step during the certification of its CRMs. As CRM producer accredited under the ISO 17034, we need our collaborators to adhere to ISO 17025 compliant requirements. Quite often, laboratories specialised in environmental or food matrices lack the necessary expertise, infrastructure/equipment or permits to process samples of human origin (that could also be potentially infectious). On the other hand, clinical laboratories typically focus on analysing biomarkers relevant to specific clinical applications, such as peptides and proteins, rather than environmental contaminants. This dichotomy creates a capacity gap in the expert laboratories community, possibly negatively impacting the development of CRMs.

Another critical challenge in the production of CRMs for HBM is procuring suitable starting material in sufficient volume. Ethical or privacy issues might complicate the process of obtaining the raw material from which to start the CRM processing. For urine, which requires non-invasive sampling, sourcing a sufficiently high amount of starting material is rather feasible, whereas this is not the case for matrices such as blood and serum. Furthermore, finding material that already contains the desired levels of environmental contaminants can be difficult. Commercial suppliers and hospitals typically do not provide detailed information on the contaminant profiles of their materials, since this is not a standard parameter in their product specifications. The possible need for fortification or spiking of the material with the target analytes is not the preferred approach for CRM producers, as it might introduce unintended alterations to the matrix, causing potential commutability issues. Moreover, in urine, many biomarkers are conjugates for which there are no commercially available analytical reference standards, for example bisphenol AF mono-β-d-glucuronide, bisphenol AF monosulfate, bisphenol B monosulfate, bisphenol Z monosulfate, and bisphenol AP monosulfate. In these cases, fortifying with parent compounds instead of metabolites would result in a material that does not reflect real-life sample. Mainly due to the lack of analytical standards and due to the fact that enzymatic deconjugation is easier and quicker, for some biomarker/matrix combinations, enzymatic deconjugation is applied in the analytical procedure, instead of quantifying the individual metabolite compounds. However, there are concerns regarding the efficiency of the enzymatic reaction, which impacts the metrological traceability of the measurement result. An appropriate validation of the analytical method by using a specific conjugate would help in properly assessing the efficiency of the enzymatic step and improving the accuracy. It has been observed that, in biomonitoring studies, important information regarding the robustness and precision of the methods used is often lacking [75]. Strikingly, it has been reported that availability of a CRM does not necessarily guarantee its utilization, thereby highlighting also a possible QA/QC-related education and awareness gap. A recent review article highlights the infrequent use of CRMs for QA/QC in analysing flame retardants in human matrices, despite their availability for serum. The importance of including evidence of the reliability of the analytical method employed might be obvious, yet it is often overlooked in practice [76]. It has been flagged that extraction recoveries are commonly reported in biomonitoring and research studies, while other important information, e.g., precision and robustness, is often lacking [75]. Funding agencies could consider giving the necessary attention in requiring that project proposals include adequate QA/QC measures, as a condition for research grant funding, especially when data collection is one of the project’s tasks. This would help in enhancing the reliability and comparability of research findings, and ultimately support the advancement of analytical and bioanalytical chemistry research.

## PFAS showcase

PFAS constitute a class of compounds that has recently attracted interest at several societal and political levels and that presents a significant analytical challenge for the scientific community. Thus, specific aspects of QA/QC will be illustrated using PFAS as a pragmatic example.

Three materials exist for the analysis of PFAS in human matrices (in serum and plasma), but no certified values are assigned for any of the PFAS analytes (Supplementary material – CRMs summary Tables S2 and S4). The explanation may be linked to the lack of high-purity, SI-traceable chemical standards for single isomeric species (e.g., linear PFOS) at the time the materials were certified. Nowadays, it is possible to purchase some metrologically traceable, high-purity single isomeric species, which are available from NIMJ. SRM 1957 and SRM 1958 greatly aid the analytical chemistry community in measuring PFAS. These materials are used as quality control materials, for interlaboratory comparison, for inter- and intra-day measurement accuracy, and as an indicator of isomeric patterns of PFAS [45]. Importantly, PFAS values are reported as the sum of both branched and linear forms of the analytes, aligning with the current US legislation requirements for the analysis of PFAS in drinking water [77–79]. With the advancement of analytical techniques, there has been a rise in the number of HBM programmes that include the measurement of isomers of the most frequently analysed PFAS (i.e., PFOS and PFOA) [61, 80].

Branched isomers may be excreted from the body more rapidly than linear isomers [81, 82]. Due to higher bioavailability, branched isomers (in comparison to linear isomers) may have higher transplacental transfer efficiencies and renal clearance, thereby being preferentially enriched in cord blood and urine [82]. These findings highlight the importance of measuring isomer-specific compounds.

A CRM in serum for legacy long-chain PFAS that includes linear and branched isomers is unequivocal needed. Novel PFAS substances, such as shorter chain and polymeric PFAS, are being released in the environment (an example of “regrettable substitution”), while longer chain PFAS (e.g., PFOA and PFOS) are slowly being banned from the market, thereby reflecting signs of decreased levels of contamination in human populations [49, 61, 83–85]. For a reliable biomonitoring of PFAS, it is crucial to develop matrix-matched RMs that include certified values for novel PFAS compounds and isomer-specific values [86, 87].

The importance of intercomparison exercises has been clearly demonstrated for PFAS measurements in serum within the HBM4EU project. Participating laboratories managed to decrease the average study relative standard deviation from 22 to 13% over all target biomarkers from the first to the last round [73]. Researchers used leftover materials from these interlaboratory comparisons after the exercise for method development for a higher number of PFAS in plasma. It is worth mentioning that this is not ideal, given the difference in matrix, which may result in possible matrix effects on the analysed compounds, as already mentioned. Yet, some methods are robust enough to be used for both serum and plasma [88].

One of the challenges in analysing human matrices such as blood, serum, and plasma, is the invasive sample collection techniques that also require skilled and authorised workers. To address this issue, a remote sampling approach was developed at Eurofins to quantify PFAS in whole blood samples collected using volumetric absorptive microsamplers (also known as dried blood spot analysis) [89].

For PFAS analysis (and applicable to other classes of compounds, e.g., phthalates), the production of a matrix RM certified for blank levels would be opportune to assist in identifying contamination issues at the laboratory’s end. Given the recent trends and results obtained in recent HBM studies, the production of such a CRM could also be a challenge, since the levels of these compounds are detectable in the majority of people’s blood [90, 91].

Hence, the analytical community is facing a challenge that is practically impossible to address using well-established, conventional tools. A known PFAS challenge is that the number of individual compounds is estimated to be more than 7 million [92]. Novel approaches, such as non-target and suspect screening, are therefore crucial for the discovery of new and unknown PFAS and their possible metabolites. The evaluation of data that combines non-target and suspect screening could help researchers identify samples that require further target analysis. The evident necessity for non-target and suspect screening applications [93, 94] urges CRM producers to provide materials designed to quality-proof results obtained using these approaches.

## Conclusions and outlook

HBM plays a vital role in assessing the potential impact of environmental chemicals on human health and in supporting public health policy developments.

These assessments are based on the availability of quality-assured and comparable HBM data. Despite growing awareness of the importance of QA/QC, this study brings data comparability among laboratories into the spotlight and shows that there is room for developing and improving options and practices. The analysis presented herein identifies present challenges and facilitates anticipation of future needs of the scientific community, particularly with regard to the role of CRMs as QA/QC tools. Out of the more than 300 biomarker-matrix combinations selected for different HBM studies worldwide (i.e., Canada, China, EU, Japan, and USA), a distilled selection has been carried out to identify the ones most needing attention in terms of QA/QC improvement. Currently, (C)RMs for many relevant biomarker-matrix combinations are lacking, such as phthalate substitutes, organophosphate pesticides, bisphenols in urine, and PFAS in serum; the JRC plans to initiate the production of dedicated CRMs. Moreover, challenges specific to the production of CRMs are discussed, such as procuring suitable starting material in relatively large volumes, and targeting desired levels of biomarkers. Furthermore, a critical examination of the availability of PT schemes, analytical methods, and CRMs, as essential QA/QC tools, has revealed their intertwined relationship reflecting gaps and challenges in the HBM field. A snapshot of HBM challenges and trends is provided through the example of PFAS. Nonlinear approaches and solutions are required to establish anchor points for reliable and metrologically traceable measurements. Collaborative efforts are needed to facilitate the advancement of HBM programmes, ultimately contributing to better-informed decision-making and policy options identification. More interaction, communication, and cooperation among entities performing HBM, PT scheme providers, academia, CRM producers, publishers, and policy makers are important components in the whole system where public health improvement emerges as a hot topic at multiple levels.

## Supplementary Information

Below is the link to the electronic supplementary material.

ESM 1 (XLSX 87.0 KB)

ESM 2 (XLSX 24.1 KB)

## Abbreviations

CHMS: Canadian Health Measures Survey
CNHBM: China National Human Biomonitoring
CRM: Certified Reference Material
CTQ: Centre de Toxicologie du Québec
CV: Coefficient of variation
EU: European Union
G-EQUAS: German External Quality Assessment Scheme
GerES: German Environmental Survey
HBM: Human biomonitoring
HBM4EU: Human Biomonitoring for Europe Joint Project
ISO: International Organization for Standardization
JECS: Japan Environment and Children’s Study
JRC: Joint Research Centre of the European Commission
KoNEHS: Korean National Environmental Health Survey
NHANES: United States National Health and Nutrition Examination Survey
NIMD: National Institute of Minamata Disease
NIST: National Institute of Standards and Technology
PAHs: Polycyclic aromatic hydrocarbons
PARC: Partnership for the Assessment of Risks in Chemicals
PBDE: Polybrominated diphenyl ether congeners
PFAS: Per- and polyfluoroalkyl substances
POPs: Persistent organic pollutants
PCBs: Polychlorinated biphenyls
PT: Proficiency testing
QA/QC: Quality assurance/quality control
SI: The International System of Units
SRM: Standard reference material
UNEP: United Nations Environment Programme
USA: United States of America
VOC: Volatile organic compounds

## Declarations

Datasets are presented in the main manuscript and additional supporting files.

## References

[1] UNEP (2019) United Nations Environment Programme, United Nations Global Chemical Outlook II.

[2] Cefic (2023) European Chemical Industry Counci. The european chemical industry a vital part of Europe’s future.

[3] EC (2020) The Europan Commission, COM(2020) 652 final, 2020/0300 (COD). Decision of the European Parliament and of the Council on a general union environment action programme to 2030.

[4] Fantke P, von Goetz N, Schluter U, Bessems J, Connolly A, Dudzina T, Ahrens A, Bridges J, Coggins MA, Conrad A, Hanninen O, Heinemeyer G, Kephalopoulos S, McLachlan M, Meijster T, Poulsen V, Rother D, Vermeire T, Viegas S, Vlaanderen J, Jeddi MZ, Bruinen de Bruin Y. Building a European exposure science strategy. J Expo Sci Environ Epidemiol. 2020;30(6):917–24. 10.1038/s41370-019-0193-7. 31792311 10.1038/s41370-019-0193-7 PMC7704392

[5] Zare Jeddi M, Hopf NB, Louro H, Viegas S, Galea KS, Pasanen-Kase R, Santonen T, Mustieles V, Fernandez MF, Verhagen H, Bopp SK, Antignac JP, David A, Mol H, Barouki R, Audouze K, Duca RC, Fantke P, Scheepers P, Ghosh M, Van Nieuwenhuyse A, Lobo Vicente J, Trier X, Rambaud L, Fillol C, Denys S, Conrad A, Kolossa-Gehring M, Paini A, Arnot J, Schulze F, Jones K, Sepai O, Ali I, Brennan L, Benfenati E, Cubadda F, Mantovani A, Bartonova A, Connolly A, Slobodnik J, Bruinen de Bruin Y, van Klaveren J, Palmen N, Dirven H, Husoy T, Thomsen C, Virgolino A, Roosli M, Gant T, von Goetz N, Bessems J. Developing human biomonitoring as a 21st century toolbox within the European exposure science strategy 2020–2030. Environ Int. 2022;168: 107476. 10.1016/j.envint.2022.107476. 36067553 10.1016/j.envint.2022.107476

[6] Schulz C, Conrad A, Becker K, Kolossa-Gehring M, Seiwert M, Seifert B. Twenty years of the German Environmental Survey (GerES): human biomonitoring-temporal and spatial (West Germany/East Germany) differences in population exposure. Int J Hyg Environ Health. 2007;210(3–4):271–97. 10.1016/j.ijheh.2007.01.034. 17347043 10.1016/j.ijheh.2007.01.034

[7] Cerna M, Krskova A, Cejchanova M, Spevackova V. Human biomonitoring in the Czech Republic: an overview. Int J Hyg Environ Health. 2012;215(2):109–19. 10.1016/j.ijheh.2011.09.007. 22014893 10.1016/j.ijheh.2011.09.007

[8] Dereumeaux C, Fillol C, Charles MA, Denys S (2017) The French human biomonitoring program: First lessons from the perinatal component and future needs. Int J Hyg Environ Health 220 (2 Pt A):64–70. 10.1016/j.ijheh.2016.11.005. 10.1016/j.ijheh.2016.11.005 27919640

[9] Pino A, Chiarotti F, Calamandrei G, Gotti A, Karakitsios S, Handakas E, Bocca B, Sarigiannis D, Alimonti A. Human biomonitoring data analysis for metals in an Italian adolescents cohort: An exposome approach. Environ Res. 2017;159:344–54. 10.1016/j.envres.2017.08.012. 28841522 10.1016/j.envres.2017.08.012

[10] Perez-Gomez B, Pastor-Barriuso R, Cervantes-Amat M, Esteban M, Ruiz-Moraga M, Aragones N, Pollan M, Navarro C, Calvo E, Roman J, Lopez-Abente G, Castano A, Bioambient.Es (2013) BIOAMBIENT.ES study protocol: rationale and design of a cross-sectional human biomonitoring survey in Spain. Environ Sci Pollut Res Int 20 (2):1193–1202. 10.1007/s11356-012-1320-3. 10.1007/s11356-012-1320-3 23184128

[11] Schoeters G, Den Hond E, Colles A, Loots I, Morrens B, Keune H, Bruckers L, Nawrot T, Sioen I, De Coster S, Van Larebeke N, Nelen V, Van de Mieroop E, Vrijens J, Croes K, Goeyens K, Baeyens W. Concept of the Flemish human biomonitoring programme. Int J Hyg Environ Health. 2012;215(2):102–8. 10.1016/j.ijheh.2011.11.006. 22178406 10.1016/j.ijheh.2011.11.006

[12] EC (2012) The European Commission. Communication from the Commission to the Council, the European Parliament, the European Economic and Social Committee - "The European Environment & Health Action Plan 2004–2010", COM(2012)673 final.

[13] Schindler BK, Esteban M, Koch HM, Castano A, Koslitz S, Canas A, Casteleyn L, Kolossa-Gehring M, Schwedler G, Schoeters G, Hond ED, Sepai O, Exley K, Bloemen L, Horvat M, Knudsen LE, Joas A, Joas R, Biot P, Aerts D, Lopez A, Huetos O, Katsonouri A, Maurer-Chronakis K, Kasparova L, Vrbik K, Rudnai P, Naray M, Guignard C, Fischer ME, Ligocka D, Janasik B, Reis MF, Namorado S, Pop C, Dumitrascu I, Halzlova K, Fabianova E, Mazej D, Tratnik JS, Berglund M, Jonsson B, Lehmann A, Crettaz P, Frederiksen H, Nielsen F, McGrath H, Nesbitt I, De Cremer K, Vanermen G, Koppen G, Wilhelm M, Becker K, Angerer J. The European COPHES/DEMOCOPHES project: towards transnational comparability and reliability of human biomonitoring results. Int J Hyg Environ Health. 2014;217(6):653–61. 10.1016/j.ijheh.2013.12.002. 24405937 10.1016/j.ijheh.2013.12.002

[14] European Human Biomonitoring Initiative. https://cordis.europa.eu/project/id/733032/reporting. Accessed Aug 2024.

[15] Partnership for the assessment of risks from chemicals. https://www.eu-parc.eu/. Accessed Aug 2024.

[16] Khaled R, Elabed S, Masarani A, Almulla A, Almheiri S, Koniyath R, Semerjian L, Abass K. Human biomonitoring of environmental contaminants in Gulf Countries - current status and future directions. Environ Res. 2023;236(Pt 1): 116650. 10.1016/j.envres.2023.116650. 37479209 10.1016/j.envres.2023.116650

[17] Zare Jeddi M, Virgolino A, Fantke P, Hopf NB, Galea KS, Remy S, Viegas S, Mustieles V, Fernandez MF, von Goetz N, Vicente JL, Slobodnik J, Rambaud L, Denys S, St-Amand A, Nakayama SF, Santonen T, Barouki R, Pasanen-Kase R, Mol HGJ, Vermeire T, Jones K, Silva MJ, Louro H, van der Voet H, Duca RC, Verhagen H, Canova C, van Klaveren J, Kolossa-Gehring M, Bessems J. A human biomonitoring (HBM) Global Registry Framework: Further advancement of HBM research following the FAIR principles. Int J Hyg Environ Health. 2021;238: 113826. 10.1016/j.ijheh.2021.113826. 34583227 10.1016/j.ijheh.2021.113826

[18] Esteban Lopez M, Goen T, Mol H, Nubler S, Haji-Abbas-Zarrabi K, Koch HM, Kasper-Sonnenberg M, Dvorakova D, Hajslova J, Antignac JP, Vaccher V, Elbers I, Thomsen C, Vorkamp K, Pedraza-Diaz S, Kolossa-Gehring M, Castano A. The European human biomonitoring platform - Design and implementation of a laboratory quality assurance/quality control (QA/QC) programme for selected priority chemicals. Int J Hyg Environ Health. 2021;234: 113740. 10.1016/j.ijheh.2021.113740. 33774419 10.1016/j.ijheh.2021.113740

[19] Joint committee for traceability in laboratory medicine https://www.jctlmdb.org/#/app/home. (accessed in August 2024).

[20] ISO International Organization for Standardization. Milk and canned evaporated milk — Determination of tin content — Spectrometric method ISO/TS 9941:2005 IDF/RM 160:2005

[21] ISO International Organization for Standardization. Milk and milk products — Determination of lead content — Graphite furnace atomic absorption spectrometric method. ISO/TS 6733:2006 IDF/RM 133:2006.

[22] ISO International Organization for Standardization. Milk and milk products — Determination of organochlorine pesticides and polychlorobiphenyls — Method using capillary gas-liquid chromatography with electron-capture detection. ISO 8260:2008 IDF 130:2008.

[23] Health Canada (2021) Sixth report on human biomonitoring of environmental chemicals in Canada. Minister of Health, Ottawa, ON. www.canada.ca/en/health-canada/services/environmental-workplacehealth/reports-publications/environmental-contaminants/sixth-report-human-biomonitoring.html. Accessed Aug 2024.

[24] CDC Centers for Disease Control and Prevention. National report on human exposure to environmental chemicals. https://www.cdc.gov/environmental-exposure-report/index.html. Accessed Aug 2024.

[25] Park B, Kim B, Kim CH, Hyun Jin Oh, Park B. Association between endocrine-disrupting chemical mixtures and non-alcoholic fatty liver disease with metabolic syndrome as a mediator among adults: A population-based study in Korea. Ecotoxicol Environ Saf. 2024;276: 116310. 10.1016/j.ecoenv.2024.116310. 38614002 10.1016/j.ecoenv.2024.116310

[26] Kinjo Y, Shibata E, Askew DJ, Tanaka R, Suga R, Shimono M, Sakuragi T, Morokuma S, Ogawa M, Sanefuji M, Hamada N, Ochiai M, Ohga S, Tsuji M, Kusuhara K, Yoshino K, Group tJECsS. Association of placental weight at birth with maternal whole blood concentration of heavy metals (cadmium, lead, mercury, selenium, and manganese): The Japan Environment and Children’s Study (JECS). Environ Int. 2024;188: 108725. 10.1016/j.envint.2024.108725. 38759546 10.1016/j.envint.2024.108725

[27] Suwannarin N, Nishihama Y, Isobe T, Nakayama SF, Group tJECsS. Urinary concentrations of environmental phenol among pregnant women in the Japan Environment and Children’s Study. Environ Int. 2024;183: 108373. 10.1016/j.envint.2023.108373. 38088018 10.1016/j.envint.2023.108373

[28] Kuraoka S, Oda M, Ohba T, Mitsubuchi H, Nakamura K, Katoh T, Group tJECsS. Association of phenol exposure during pregnancy and asthma development in children: The Japan Environment and Children’s study. Environ Pollut. 2024;361: 124801. 10.1016/j.envpol.2024.124801. 39181305 10.1016/j.envpol.2024.124801

[29] Nakayama SF, Isobe T, Iwai-Shimada M, Kobayashi Y, Nishihama Y, Taniguchi Y, Sekiyama M, Michikawa T, Yamazaki S, Nitta H, Oda M, Mitsubuchi H, Sanefuji M, Ohga S, Mise N, Ikegami A, Suga R, Shimono M. Poly- and perfluoroalkyl substances in maternal serum: Method development and application in Pilot Study of the Japan Environment and Children’s Study. J Chromatogr A. 2020;1618: 460933. 10.1016/j.chroma.2020.460933. 32035665 10.1016/j.chroma.2020.460933

[30] Nishihama Y, Nakayama SF, Isobe T, Kamijima M, Group tJECsS. Association between maternal urinary neonicotinoid concentrations and child development in the Japan Environment and Children’s Study. Environ Int. 2023;181: 108267. 10.1016/j.envint.2023.108267. 37864904 10.1016/j.envint.2023.108267

[31] Nishihama Y, Nakayama SF, Isobe T, Jung CR, Iwai-Shimada M, Kobayashi Y, Michikawa T, Sekiyama M, Taniguchi Y, Yamazaki S, Group tJEaCsS (2021) Urinary metabolites of organophosphate pesticides among pregnant women participating in the Japan Environment and Children’s Study (JECS). Int J Environ Res Public Health 18 (11). 10.3390/ijerph18115929. 10.3390/ijerph18115929 PMC8199379 34073036

[32] Cao Z, Lin S, Zhao F, Lv Y, Qu Y, Hu X, Yu S, Song S, Lu Y, Yan H, Liu Y, Ding L, Zhu Y, Liu L, Zhang M, Wang T, Zhang W, Fu H, Jin Y, Cai J, Zhang X, Yan C, Ji S, Zhang Z, Dai J, Zhu H, Gao L, Yang Y, Li C, Zhou J, Ying B, Zheng L, Kang Q, Hu J, Zhao W, Zhang M, Yu X, Wu B, Zheng T, Liu Y, Barry Ryan P, Barr DB, Qu W, Zheng Y, Shi X. Cohort profile: China National Human Biomonitoring (CNHBM)-A nationally representative, prospective cohort in Chinese population. Environ Int. 2021;146: 106252. 10.1016/j.envint.2020.106252. 33242729 10.1016/j.envint.2020.106252 PMC7828642

[33] European proficiency testing information system https://www.eptis.org/. (accessed in August 2024 ).

[34] WHO (2023) World Health Organizatio, Copenhagen: WHO Regional Office for Europen. Human biomonitoring programmes: importance for protecting human health from negative impacts of chemicals. Technical summary.

[35] EC (2023) The European Commission. Commission regulation (EU) 2023/915 of 25 April 2023 on maximum levels for certain contaminants in food and repealing Regulation (EC) No 1881/2006.

[36] FAO (2023) Food and Agriculture Organization of the United Nations. General standard for contaminats and toxins in food and feed cxs 193–1995.

[37] EC (2000) The European Commission. Directive 2000/60/EC of the European Parliament and of the Council 23 October 2000.

[38] Vorkamp K, Castano A, Antignac JP, Boada LD, Cequier E, Covaci A, Esteban Lopez M, Haug LS, Kasper-Sonnenberg M, Koch HM, Perez Luzardo O, Osite A, Rambaud L, Pinorini MT, Sabbioni G, Thomsen C. Biomarkers, matrices and analytical methods targeting human exposure to chemicals selected for a European human biomonitoring initiative. Environ Int. 2021;146: 106082. 10.1016/j.envint.2020.106082. 33227583 10.1016/j.envint.2020.106082

[39] Lee S, Ahn RM, Kim JH, Han YD, Lee JH, Son BS, Lee K (2019) Study design, rationale and procedures for human biomonitoring of hazardous chemicals from foods and cooking in Korea. Int J Environ Res Public Health 16 (14). 10.3390/ijerph16142583. 10.3390/ijerph16142583 PMC6678262 31331024

[40] CDC Centers for Disease Control and Prevention. National report on human exposure to environmental chemicals. Chemical selection. https://www.cdc.gov/environmental-exposure-report/about-the-chemicals/index.html. Accessed Aug 2024.

[41] Liem AK, Furst P, Rappe C. Exposure of populations to dioxins and related compounds. Food Addit Contam. 2000;17(4):241–59. 10.1080/026520300283324. 10912239 10.1080/026520300283324

[42] Freire C, Iribarne-Duran LM, Gil F, Olmedo P, Serrano-Lopez L, Pena-Caballero M, Hurtado JA, Alvarado-Gonzalez NE, Fernandez MF, Peinado FM, Artacho-Cordon F, Olea N. Concentrations and determinants of lead, mercury, cadmium, and arsenic in pooled donor breast milk in Spain. Int J Hyg Environ Health. 2022;240: 113914. 10.1016/j.ijheh.2021.113914. 34974272 10.1016/j.ijheh.2021.113914

[43] Guerranti C, Palmieri M, Mariottini M, Focardi SE. Persistent organic pollutants in human milk from central Italy: levels and time trends. ISRN Toxicol. 2011;2011: 107514. 10.5402/2011/107514. 23724278 10.5402/2011/107514 PMC3658789

[44] Linsinger T, Auclair G, Deprez L. Commutability study on three CRMs evaluating their suitability for calibration, trueness verification and statistical quality control of methods measuring metal concentrations in human blood. Anal Bioanal Chem. 2025. 10.1007/s00216-025-05751-0. 39904899 10.1007/s00216-025-05751-0 PMC12003512

[45] Rodowa AE, Reiner JL. Utilization of a NIST SRM: a case study for per- and polyfluoroalkyl substances in NIST SRM 1957 organic contaminants in non-fortified human serum. Anal Bioanal Chem. 2021;413(9):2295–301. 10.1007/s00216-021-03241-7. 33651119 10.1007/s00216-021-03241-7 PMC8371558

[46] ISO International Organization for Standardization. Medical devices — Quality management systems — Requirements for regulatory purposes. ISO 13485:2016.

[47] Haines DA, Saravanabhavan G, Werry K, Khoury C (2017) An overview of human biomonitoring of environmental chemicals in the Canadian Health Measures Survey: 2007–2019. Int J Hyg Environ Health 220 (2 Pt A):13–28. 10.1016/j.ijheh.2016.08.002. 10.1016/j.ijheh.2016.08.002 27601095

[48] Poothong S, Thomsen C, Padilla-Sanchez JA, Papadopoulou E, Haug LS. Distribution of novel and well-known poly- and perfluoroalkyl substances (PFASs) in human serum, plasma, and whole blood. Environ Sci Technol. 2017;51(22):13388–96. 10.1021/acs.est.7b03299. 29056041 10.1021/acs.est.7b03299

[49] Schoeters G, Govarts E, Bruckers L, Den Hond E, Nelen V, De Henauw S, Sioen I, Nawrot TS, Plusquin M, Vriens A, Covaci A, Loots I, Morrens B, Coertjens D, Van Larebeke N, De Craemer S, Croes K, Lambrechts N, Colles A, Baeyens W (2017) Three cycles of human biomonitoring in Flanders - Time trends observed in the Flemish Environment and Health Study. Int J Hyg Environ Health 220 (2 Pt A):36–45. 10.1016/j.ijheh.2016.11.006. 10.1016/j.ijheh.2016.11.006 28160993

[50] Haervig KK, Petersen KU, Giwercman A, Hougaard KS, Hoyer BB, Lindh C, Ramlau-Hansen CH, Nybo Andersen AM, Toft G, Bonde JP, Tottenborg SS. Fetal exposure to maternal cigarette smoking and male reproductive function in young adulthood. Eur J Epidemiol. 2022;37(5):525–38. 10.1007/s10654-022-00869-2. 35476275 10.1007/s10654-022-00869-2

[51] Hausken T, Skaare JU, Polder A, Haugen M, Meltzer HM, Lundebye AK, Julshamn K, Nygard O, Berge RK, Skorve J. High consumption of farmed salmon does not disrupt the steady state of persistent organic pollutants (POP) in human plasma and adipose tissue. J Toxicol Environ Health A. 2014;77(20):1229–50. 10.1080/15287394.2014.926262. 25208663 10.1080/15287394.2014.926262

[52] Li Y, Huang YS, He B, Liu R, Qu G, Yin Y, Shi J, Hu L, Jiang G. Cadmium-binding proteins in human blood plasma. Ecotoxicol Environ Saf. 2020;188: 109896. 10.1016/j.ecoenv.2019.109896. 31704329 10.1016/j.ecoenv.2019.109896

[53] Hinners TA, Terrill WJ, Kent JL. Hair-metal binding. Envrironmental Health Perspectives. 1974;8:191–9. 10.1289/ehp.748191 PMC1474933 4377869

[54] Olmedo P, Pla A, Hernandez AF, Lopez-Guarnido O, Rodrigo L, Gil F. Validation of a method to quantify chromium, cadmium, manganese, nickel and lead in human whole blood, urine, saliva and hair samples by electrothermal atomic absorption spectrometry. Anal Chim Acta. 2010;659(1–2):60–7. 10.1016/j.aca.2009.11.056. 20103106 10.1016/j.aca.2009.11.056

[55] Tehrani MW, Yang KX, Parsons PJ. Development and characterization of reference materials for trace element analysis of keratinized matrices. Anal Bioanal Chem. 2020;412(8):1847–61. 10.1007/s00216-020-02432-y. 32020317 10.1007/s00216-020-02432-y PMC7197407

[56] Marín-Sáez J, López-Ruiz R, Sobral M, Romero-González R, Garrido Frenich A, Ferreira IMPLVO (2023) Analytical methods for biomonitoring organic chemical hazards in saliva: A systematic review. TrAC Trends in Analytical Chemistry 158. 10.1016/j.trac.2022.116853.

[57] Quebec National Institute of Public Health. External quality assessment schemes. https://www.inspq.qc.ca/ctq/paqe. Accessed Aug 2024.

[58] German external quality assessment scheme. https://app.g-equas.de/web/. Accessed Aug 2024.

[59] United Nations Environment Programme. Global mercury monitoring. https://www.unep.org/global-mercury-monitoring?_ga=2.26018494.2000228989.1729846356-781588227.1729846356. Accessed Aug 2024.

[60] de Boer J, van der Veen I, Fiedler H. Global interlaboratory assessments on PCBs, organochlorine pesticides and brominated flame retardants in various environmental matrices 2017/2019. Chemosphere. 2022;295: 133991. 10.1016/j.chemosphere.2022.133991. 35167837 10.1016/j.chemosphere.2022.133991

[61] Fiedler H, Sadia M, Krauss T, Baabish A, Yeung LWY (2022) Perfluoroalkane acids in human milk under the global monitoring plan of the Stockholm Convention on Persistent Organic Pollutants (2008–2019). Frontiers of Environmental Science & Engineering 16 (10). 10.1007/s11783-022-1541-8.

[62] Drash G, Roider G. Assessment of hair mineral analysis commercially offered in Germany. J Trace Elem Med Biol. 2002;16:27–31. 11878749 10.1016/S0946-672X(02)80005-0

[63] Skroder H, Kippler M, Nermell B, Tofail F, Levi M, Rahman SM, Raqib R, Vahter M. Major limitations in using element concentrations in hair as biomarkers of exposure to toxic and essential trace elements in children. Environ Health Perspect. 2017;125(6): 067021. 10.1289/EHP1239. 28669939 10.1289/EHP1239 PMC5743543

[64] Jursa T, Stein CR, Smith DR (2018) Determinants of hair manganese, lead, cadmium and arsenic levels in environmentally exposed children. Toxics 6 (2). 10.3390/toxics6020019. 10.3390/toxics6020019 PMC6027252 29565296

[65] Rodushkin I, Axelsson MD (2003) Application of double focusing sector field ICP-MS for multielemental characterization of human hair and nails. Part III. Direct analysis by laser ablation. Sci Total Environ 305 (1–3):23–39. 10.1016/S0048-9697(02)00463-1. 10.1016/S0048-9697(02)00463-1 12670755

[66] Namkoong S, Hong SP, Kim MH, Park BC. Reliability on intra-laboratory and inter-laboratory data of hair mineral analysis comparing with blood analysis. Ann Dermatol. 2013;25(1):67–72. 10.5021/ad.2013.25.1.67. 23467102 10.5021/ad.2013.25.1.67 PMC3582931

[67] Pozebon D, Scheffler GL, Dressler VL. Elemental hair analysis: A review of procedures and applications. Anal Chim Acta. 2017;992:1–23. 10.1016/j.aca.2017.09.017. 29054142 10.1016/j.aca.2017.09.017

[68] Han X, Chen H, Shen M, Deng M, Du B, Zeng L. Hair and nails as noninvasive bioindicators of human exposure to chlorinated paraffins: Contamination patterns and potential influencing factors. Sci Total Environ. 2021;798: 149257. 10.1016/j.scitotenv.2021.149257. 34315053 10.1016/j.scitotenv.2021.149257

[69] UNEP United Nations Environment Programme. POPs interlaboratory assessments. https://www.unep.org/pops-interlaboratory-assessments. Accessed Aug 2024.

[70] Mol HGJ, Elbers I, Palmke C, Bury D, Goen T, Lopez ME, Nubler S, Vaccher V, Antignac JP, Dvorakova D, Hajslova J, Sakhi AK, Thomsen C, Vorkamp K, Castano A, Koch HM (2022) Proficiency and interlaboratory variability in the determination of phthalate and DINCH biomarkers in human urine: Results from the HBM4EU Project. Toxics 10 (2). 10.3390/toxics10020057. 10.3390/toxics10020057 PMC8878211 35202244

[71] Galusha AL, Merrill L, Palmer CD, Amarasiriwardena C, Parsons PJ. Measurement harmonization and traceability for trace element analyses across the Children’s Health Exposure Analysis Resource laboratory network. Environ Res. 2021;193: 110302. 10.1016/j.envres.2020.110302. 33049243 10.1016/j.envres.2020.110302 PMC8924990

[72] Nubler S, Lopez ME, Castano A, Mol H, Schafer M, Haji-Abbas-Zarrabi K, Bury D, Koch HM, Vaccher V, Antignac JP, Dvorakova D, Hajslova J, Thomsen C, Vorkamp K, Goen T. Interlaboratory comparison investigations (ICI) and external quality assurance schemes (EQUAS) for cadmium in urine and blood: Results from the HBM4EU project. Int J Hyg Environ Health. 2021;234: 113711. 10.1016/j.ijheh.2021.113711. 33714064 10.1016/j.ijheh.2021.113711

[73] Nubler S, Esteban Lopez M, Castano A, Mol HGJ, Haji-Abbas-Zarrabi K, Schafer M, Muller J, Hajslova J, Dvorakova D, Antignac JP, Koch HM, Haug LS, Vorkamp K, Goen T. Interlaboratory Comparison Investigations (ICIs) and External Quality Assurance Schemes (EQUASs) for human biomonitoring of perfluoroalkyl substances (PFASs) in serum as part of the quality assurance programme under HBM4EU. Sci Total Environ. 2022;847: 157481. 10.1016/j.scitotenv.2022.157481. 35868372 10.1016/j.scitotenv.2022.157481

[74] PARC Selected biomarkers of exposure for PARC Aligned Studies. 2024. https://www.eu-parc.eu/sites/default/files/2024-03/PARC%20selected%20exposure%20biomarkers%20for%20Aligned%20Studies_v4.pdf. Accessed August 2024.

[75] Hajeb P, Castano A, Cequier E, Covaci A, Lopez ME, Antuna AG, Haug LS, Henriquez-Hernandez LA, Melymuk L, Perez Luzardo O, Thomsen C, Vorkamp K. Critical review of analytical methods for the determination of flame retardants in human matrices. Anal Chim Acta. 2022;1193: 338828. 10.1016/j.aca.2021.338828. 35058002 10.1016/j.aca.2021.338828

[76] Wise SA. What if using certified reference materials (CRMs) was a requirement to publish in analytical/bioanalytical chemistry journals? Anal Bioanal Chem. 2022;414(24):7015–22. 10.1007/s00216-022-04163-8. 35697811 10.1007/s00216-022-04163-8

[77] EC (2020) The European Commission. Directive (EU) 2020/2184 of the European Parliament and of the Council of 16 December 2020 on the quality of water intended for human consumption.

[78] Shoemaker JA, Grimmett P, B. B,. Determination of selected perfluorinated alkyl acids in drinking water by solid phase extraction and liquid chromatography/tandem mass spectrometry (LC/MS/MS). Washington, DC: US Environmental Protection Agency; 2008.

[79] Rosenblum L, Wendelken SC (2019) Determination of per- and polyfluoroalkyl substances in drinking water by isotope dilution anion exchange solid phase extraction and liquid chromatography/tandem mass spectrometry. United States Environmental Protection Agency

[80] Toms LML, Braunig J, Vijayasarathy S, Phillips S, Hobson P, Aylward LL, Kirk MD, Mueller JF. Per- and polyfluoroalkyl substances (PFAS) in Australia: Current levels and estimated population reference values for selected compounds. Int J Hyg Environ Health. 2019;222(3):387–94. 10.1016/j.ijheh.2019.03.004. 30898527 10.1016/j.ijheh.2019.03.004

[81] Benskin JP, Holt A, Martin JW. Isomer-specific biotransformation rates of a perfluorooctane sulfonate (PFOS)-precursor by cytochrome P450 isozymes and human liver microsomes. Environ Sci Technol. 2009;43:8566–72. 20028053 10.1021/es901915f

[82] Beesoon S, Martin JW. Isomer-Specific Binding Affinity of Perfluorooctanesulfonate (PFOS) and Perfluorooctanoate (PFOA) to Serum Proteins. Environ Sci Technol. 2015;49(9):5722–31. 10.1021/es505399w. 25826685 10.1021/es505399w

[83] Sochorova L, Hanzlikova L, Cerna M, Drgacova A, Fialova A, Svarcova A, Gramblicka T, Pulkrabova J (2017) Perfluorinated alkylated substances and brominated flame retardants in serum of the Czech adult population. Int J Hyg Environ Health 220 (2 Pt A):235–243. 10.1016/j.ijheh.2016.09.003 10.1016/j.ijheh.2016.09.003 27743851

[84] Sundstrom M, Ehresman DJ, Bignert A, Butenhoff JL, Olsen GW, Chang SC, Bergman A. A temporal trend study (1972–2008) of perfluorooctanesulfonate, perfluorohexanesulfonate, and perfluorooctanoate in pooled human milk samples from Stockholm. Sweden Environ Int. 2011;37(1):178–83. 10.1016/j.envint.2010.08.014. 20880590 10.1016/j.envint.2010.08.014

[85] Pollock T, Karthikeyan S, Walker M, Werry K, St-Amand A. Trends in environmental chemical concentrations in the Canadian population: Biomonitoring data from the Canadian Health Measures Survey 2007–2017. Environ Int. 2021;155: 106678. 10.1016/j.envint.2021.106678. 34118655 10.1016/j.envint.2021.106678

[86] Fiedler H, van der Veen I, de Boer J (2020) Global interlaboratory assessments of perfluoroalkyl substances under the Stockholm Convention on persistent organic pollutants. TrAC Trends in Analytical Chemistry 124. 10.1016/j.trac.2019.03.023

[87] Jahnke A, Berger U. Trace analysis of per- and polyfluorinated alkyl substances in various matrices-How do current methods perform? J Chromatogr A. 2009;1216(3):410–21. 10.1016/j.chroma.2008.08.098. 18817914 10.1016/j.chroma.2008.08.098

[88] Frigerio G, Cafagna S, Polledri E, Mercadante R, Fustinoni S. Development and validation of an LC-MS/MS method for the quantitation of 30 legacy and emerging per- and polyfluoroalkyl substances (PFASs) in human plasma, including HFPO-DA, DONA, and cC6O4. Anal Bioanal Chem. 2022;414(3):1259–78. 10.1007/s00216-021-03762-1. 34907451 10.1007/s00216-021-03762-1 PMC8760233

[89] Carignan CC, Bauer RA, Patterson A, Phomsopha T, Redman E, Stapleton HM, Higgins CP. Self-Collection Blood Test for PFASs: Comparing Volumetric Microsamplers with a Traditional Serum Approach. Environ Sci Technol. 2023;57(21):7950–7. 10.1021/acs.est.2c09852. 37189231 10.1021/acs.est.2c09852 PMC10233751

[90] Rodriguez-Carrillo A, Salamanca-Fernandez E, den Hond E, Verheyen VJ, Fabelova L, Murinova LP, Pedraza-Diaz S, Castano A, Garcia-Lario JV, Remy S, Govarts E, Schoeters G, Olea N, Freire C, Fernandez MF. Association of exposure to perfluoroalkyl substances (PFAS) and phthalates with thyroid hormones in adolescents from HBM4EU aligned studies. Environ Res. 2023;237(Pt 1): 116897. 10.1016/j.envres.2023.116897. 37598845 10.1016/j.envres.2023.116897

[91] Shearer JJ, Callahan CL, Calafat AM, Huang WY, Jones RR, Sabbisetti VS, Freedman ND, Sampson JN, Silverman DT, Purdue MP, Hofmann JN. Serum Concentrations of Per- and Polyfluoroalkyl Substances and Risk of Renal Cell Carcinoma. J Natl Cancer Inst. 2021;113(5):580–7. 10.1093/jnci/djaa143. 32944748 10.1093/jnci/djaa143 PMC8096365

[92] Schymanski EL, Zhang J, Thiessen PA, Chirsir P, Kondic T, Bolton EE. Per- and polyfluoroalkyl substances (PFAS) in PubChem: 7 million and growing. Environ Sci Technol. 2023;57(44):16918–28. 10.1021/acs.est.3c04855. 37871188 10.1021/acs.est.3c04855 PMC10634333

[93] Megson D, Niepsch D, Spencer J, Santos CD, Florance H, MacLeod CL, Ross I. Non-targeted analysis reveals hundreds of per- and polyfluoroalkyl substances (PFAS) in UK freshwater in the vicinity of a fluorochemical plant. Chemosphere. 2024;367: 143645. 10.1016/j.chemosphere.2024.143645. 39476983 10.1016/j.chemosphere.2024.143645

[94] Koelmel JP, Lin EZ, Parry E, Stelben P, Rennie EE, Godri Pollitt KJ. Novel perfluoroalkyl substances (PFAS) discovered in whole blood using automated non-targeted analysis of dried blood spots. Sci Total Environ. 2023;883: 163579. 10.1016/j.scitotenv.2023.163579. 37100129 10.1016/j.scitotenv.2023.163579 PMC10247435

